# Identification of Novel Antagonists Targeting Cannabinoid Receptor 2 Using a Multi-Step Virtual Screening Strategy

**DOI:** 10.3390/molecules26216679

**Published:** 2021-11-04

**Authors:** Mukuo Wang, Shujing Hou, Ye Liu, Dongmei Li, Jianping Lin

**Affiliations:** 1 State Key Laboratory of Medicinal Chemical Biology, College of Pharmacy, Tianjin Key Laboratory of Molecular Drug Research, Nankai University, Haihe Education Park, 38 Tongyan Road, Tianjin 300350, China; wangmukuo@126.com (M.W.); 2120191123@mail.nankai.edu.cn (S.H.); 2120191125@mail.nankai.edu.cn (Y.L.); 2 Biodesign Center, Tianjin Institute of Industrial Biotechnology, Chinese Academy of Sciences, 32 West 7th Avenue, Tianjin Airport Economic Area, Tianjin 300308, China; 3 Platform of Pharmaceutical Intelligence, Tianjin International Joint Academy of Biomedicine, Tianjin 300457, China

**Keywords:** CB2 receptor antagonist, deep learning, pharmacophore, molecular docking, multi-step virtual screening

## Abstract

The endocannabinoid system plays an essential role in the regulation of analgesia and human immunity, and Cannabinoid Receptor 2 (CB2) has been proved to be an ideal target for the treatment of liver diseases and some cancers. In this study, we identified CB2 antagonists using a three-step “deep learning–pharmacophore–molecular docking” virtual screening approach. From the ChemDiv database (1,178,506 compounds), 15 hits were selected and tested by radioligand binding assays and cAMP functional assays. A total of 7 out of the 15 hits were found to exhibit binding affinities in the radioligand binding assays against CB2 receptor, with a pK_i_ of 5.15–6.66, among which five compounds showed antagonistic activities with pIC_50_ of 5.25–6.93 in the cAMP functional assays. Among these hits, Compound 8 with the 4H-pyrido[1,2-a]pyrimidin-4-one scaffold showed the best binding affinity and antagonistic activity with a pK_i_ of 6.66 and pIC_50_ of 6.93, respectively. The new scaffold could serve as a lead for further development of CB2 drugs. Additionally, we hope that the model in this study could be further utilized to identify more novel CB2 receptor antagonists, and the developed approach could also be used to design potent ligands for other therapeutic targets.

## 1. Introduction

The G-protein-coupled receptors (GPCRs), containing about 800 members, are the largest membrane protein family known in the human body [1]. GPCRs are the largest class of drug targets. About one-third of the marketed drugs exert their effects by binding to GPCRs and modifying their intracellular signals [2]. Cannabinoid (CB) receptors are one of the members of the GPCR family, with two discovered isoforms, i.e., CB1 and CB2, which were cloned in 1990 and 1993, respectively [3,4]. CB1 and CB2 receptors bind to adenylate cyclase and mitogen-activated protein kinase through G protein (mainly through coupling with the G_i_/G_o_ protein) [5]. The CB1 receptor is also coupled to several calcium and potassium channels via G protein [6]. These receptors are widely distributed in the central and peripheral neurons, and inhibition of neurotransmitter release is their main function. The CB2 receptor mainly distributes in immune cells, whereas the surface of these immune cells also contains the CB1 receptor. Although the expression of the CB1 receptor is low, both receptors exert a wide range of immune functions, such as regulating the release of cytokines [7]. In addition, the CB2 receptor is also slightly expressed in the central nervous system [8].

The CB2 receptor has proved to be an ideal target for immunomodulation, pain management, osteoporosis, and liver disease treatment [9,10,11]. Zhou et al. showed that inhibition of the CB2 receptor could improve renal fibrosis [12]. In addition, Xiang et al. discovered that the CB2 receptor might become a potential target for the treatment of genetic cancer, so CB2 receptor antagonists could act as a potential drug for cancer treatment [13]. Therefore, the identification and design of CB2 receptor antagonists are necessary and important.

**Figure 1:**
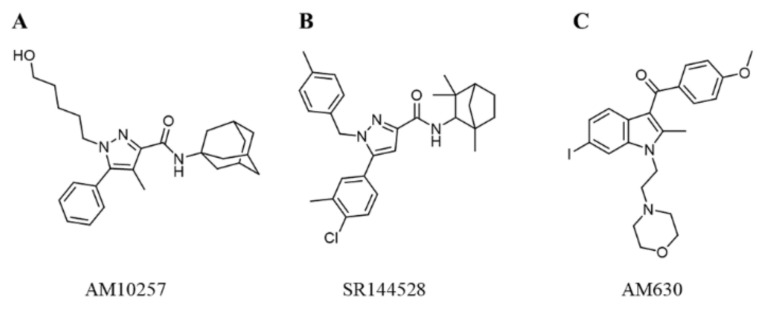
Representative chemical structures of known CB2 receptor antagonists: (**A**) AM10257, (**B**) SR144528, and (**C**) AM630.

In 2019, Li et al. [14] designed a high-affinity, CB2-receptor-specific antagonist AM10257 (Figure 1A) through systematic optimization of the CB1 receptor antagonist SR141716A (Rimonabant) [15], and the crystal structure of CB2 receptor in complex with AM10257 was resolved with a resolution of 2.80 Å. The overall structure of the CB2 receptor consists of a 7TM bundle (helix I-VII) and an intracellular double-channel helix VIII. The antagonist AM10257 resides in the orthosteric ligand binding site of the CB2 receptor, which contains key residues Phe183^ECL2^, Phe87^2.57^, Val113^3.32^, Phe117^3.36^, Trp258^6.48^, Thr114^3.33^, Phe183^ECL2^, Ile186^ECL2^, and Trp194^5.43^, Ser165^4.57^, Phe87^2.57^, Phe91^2.61^, Phe94^2.64^, His95^2.65^, and Phe183^ECL2^. This will facilitate the design of CB2 receptor antagonists dramatically.

In the past decade, many CB2 receptor antagonists have been discovered. Rinaldi-Carmona et al. introduced the first, highly potent, selective, and orally active CB2 receptor antagonist SR144528 (pK_i_ = 8.3–9.2) (Figure 1B) [16]. Pertwee et al. identified AM630 as a selective CB2 receptor antagonist with a pK_i_ value of 7.5 (Figure 1C) [17]. Until now, there are 4250 compounds with K_i_ values that have been deposited in the ChEMBL27 database [18] against the CB2 receptor. However, none of the existing CB2 receptor antagonists have entered the clinic.

In recent years, numerous in silico methods have been applied to discover and develop new CB2 receptor antagonists. Markt et al. developed pharmacophore models based on CB2 receptor antagonists and identified seven compounds that showed K_i_ values of <25 μM [19]. Chen et al. [20] constructed a 3D CB2 receptor homology structure model based on the crystal structure of bovine rhodopsin. Based on the homology structure, they established a virtual screening protocol, which successfully distinguished known bioactive CB2 receptor antagonists from randomly chosen molecules. Hu et al. [21] constructed models of both inactive and active CB2 receptors by homology modeling, based on which they identified 10 hits targeting the CB2 receptor with a K_i_ of <3 μM using pharmacophore modeling, docking, and experimental validations. However, the in silico methods need to be improved since the binding affinities of most of the identified CB2 antagonists are still low.

In this study, we identified CB2 receptor antagonists using a developed multi-step virtual screening strategy integrating deep learning (including deep neural networks, DNNs, and convolutional neural networks, CNNs), pharmacophore, and molecular docking methods [22] (a “deep learning–pharmacophore–molecular docking” virtual screening workflow). By screening the ChemDiv database (1,178,506 compounds), 13 of 15 selected hits were found to exhibit binding affinity in the radioligand binding assays, among which seven compounds showed pK_i_ values of 5.15–6.66. In the following cAMP functional assays, five of the seven compounds showed antagonistic activity against the CB2 receptor with a pIC_50_ of 5.25–6.93. Among these compounds, Compound 8 with the 4H-pyrido[1,2-a]pyrimidin-4-one scaffold showed the highest binding affinity (pK_i_ = 6.66) and the most potent antagonistic activity (pIC_50_ = 6.93) towards the CB2 receptor.

## 2. Results

### 2.1. Construction and Validation of DNN and CNN Models

The DNN and CNN models were constructed using the extended connectivity fingerprint 4 (ECFP4) [23] and neural fingerprint (NFP) [24], respectively. In order to find a batch size that maximizes the performance of the DNN model, we constructed seven CB2-receptor-antagonist DNN classification models with batch sizes ranging from 50 to 350 with an interval of 50, which were tested on the test set. Table 1 shows the results. From Table 1, we can see that Model_D6 with a batch size of 300 shows the best prediction ability, with an AUC of 0.986 and an MCC of 0.917, and the SE, SP, Q+, and Q− of Model_D6 are also better than those of the other models.

The performance of the CNN models was also evaluated on batch sizes from 50 to 400 with an interval of 50. Table 2 shows the performance of 8 CNN models. Model_C7 with a batch size of 350 shows the best prediction ability, with an AUC of 0.967 and an MCC of 0.860, and the SE, SP, Q+, and Q- of Model_C7 are also better than those of the other models. Based on the performance results of DNN and CNN models, we selected Model_D6 and Model_C7 for further screening.

### 2.2. Generation of Pharmacophore Models

**Figure 2:**
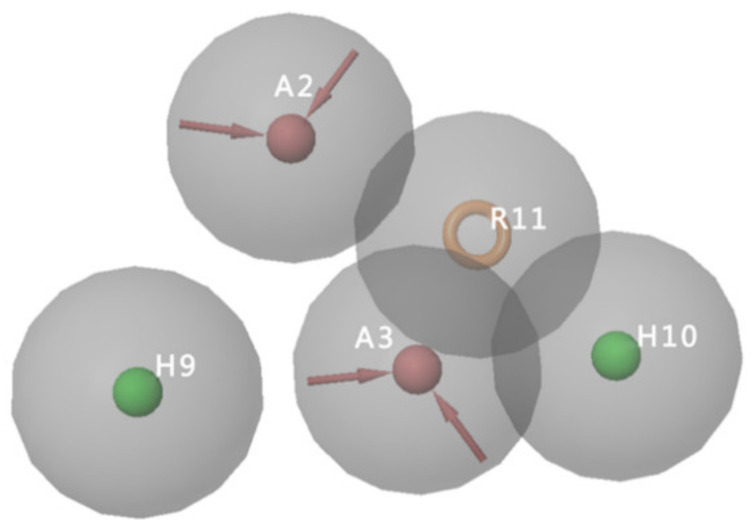
Pharmacophore features of pharmacophore hypotheses AAHHR_4.

The pharmacophore models of the CB2 receptor antagonists were generated based on a training set of eight biologically active CB2 antagonists (Table S1). Twenty pharmacophore hypotheses matching four to eight of the eight activities were generated. A validation set consisting of 29 CB2 receptor antagonists and 858 decoys was used to evaluate the pharmacophore hypotheses. Table 3 summarizes the validation results of the 20 pharmacophore models. Model AAHHR_4, with PhaseHypoScore of 0.85, EF1% of 13.59, BEDROC of 0.47, ROC of 0.68, and AUAC of 0.68, shows the best performance, which matches the eight active molecules in the training set. Figure 2 shows the pharmacophore features of the pharmacophore hypotheses AAHHR_4, which includes two hydrogen bond acceptors (A), two hydrophobic groups (H), and one aromatic ring (R). Hence, the AAHHR_4 model (Figure 2) was selected for further virtual screening.

### 2.3. Virtual Screening

**Figure 3:**
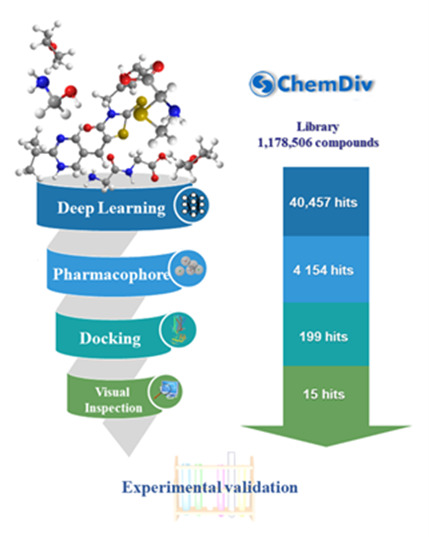
Workflow of the multi-step virtual screening of the ChemDiv database targeting CB2 receptors.

Our previous study [22] showed that a multi-step virtual screening including deep learning (DNN and CNN) classification models, pharmacophore models, and molecular docking efficiently identifies dual A_1_/A_2A_ AR antagonists. In this study, we used the same strategy to discover CB2 receptor antagonists. Figure 3 shows the schematic workflow of the multi-step virtual screening. In the first step, the selected DNN and CNN models were used to screen the 1,178,506 compounds in the ChemDiv library. With the DNN and CNN models, 181,490 and 153,438 compounds passed the filter, respectively. A total of 40,457 compounds were predicted to be active by both DNN and CNN models, which were retained for the next step. In the second step, the AAHHR_4 pharmacophore model was used to screen the 40,457 compounds. A total of 4154 of the 40,457 compounds that match at least four of the five characteristic points were retained. In the third step, the 4154 compounds were docked to the X-ray structure of the CB2 receptor (PDB ID 5ZTY) at Glide HTVS, SP, and XP levels, sequentially. A total of 199 compounds were retained after XP molecular docking. Finally, 15 compounds were selected with visual inspection and proceeded for further in vitro biological activity evaluation against the CB2 receptor.

### 2.4. Biological Evaluation

#### 2.4.1. CB2 Receptor Affinity Experiments

Table 4 summarizes the obtained binding affinities of the 15 hits (No. 1–15) from multi-step virtual screening in radioligand binding assays at the human CB2 receptor with the CB2 receptor antagonist WIN-55212-2 as a control. Table 4 shows that 13 of 15 compounds (“hits rate” of 86.7%) showed activity of pK_i_ > 4.4 towards the CB2 receptor (Figure S1). A total of 7 compounds (Compounds 1, 3, 6, 7, 8, 12, 15) had a micromolar affinity for the CB2 receptor with a pK_i_ value of 5.15–6.66, among which Compound 8 showed a nanomolar affinity for the CB2 receptor (pK_i_ = 6.66). The concentration–response curves of the seven compounds are shown in Figure S1.

#### 2.4.2. CB2 Receptor Functional Experiments

Compounds 1, 3, 6, 7, 8, 12, and 15 were further evaluated by cAMP functional assays. Figure S2 presents the concentration–response curves of the seven compounds against the CB2 receptor in the cAMP functional assays. A total of 5 compounds (Compounds 3, 7, 8, 12, and 15) possessed antagonistic activity toward the CB2 receptor with pIC_50_ values of 5.25–6.93 (Table 4). Notably, Compound 8 has the most potent antagonistic activity towards the CB2 receptor with a pIC_50_ value of 6.93, which is consistent with the highest binding affinity for the CB2 receptor with a pK_i_ value of 6.66.

#### 2.4.3. Selectivity of the Hit Compounds for the CB2 Receptor over the CB1 Receptor

The binding and functional selectivity of the five compounds were tested by performing radioligand binding assays and cAMP functional assays against the CB1 receptor. The five compounds (Compounds 3, 7, 8, 12, and 15) possessed weak binding affinity against the CB1 receptor, compared with those against the CB2 receptor, with pK_i_ values of 4.42–5.47 in the radioligand binding assays (Table S2 and Figure S3). Among them, three compounds (Compounds 3, 8, and 12) showed more than 20 micromolar antagonistic activities (pIC_50_ < 4.7) against the CB1 receptor (Table S2 and Figure S4). Compared with the pK_i_ values of 5.25–6.47 against the CB2 receptor, these three compounds (Compounds 3, 8, and 12, with a pIC_50_ < 4.7) showed good selectively for the CB2 receptor over the CB1 receptor in the cAMP functional assays. Specifically, Compound 8 exhibited more than 100 times the antagonistic activity towards the CB2 receptor (pIC_50_ value of 6.93) in comparison to the CB1 receptor (pIC_50_ < 4.70).

### 2.5. Novelty Analysis of Hit Compounds

To further evaluate the novelty of the compounds (Compounds 3, 7, 8, 12, and 15), the pairwise similarity between the tested compounds and all the known CB2 receptor antagonists in ChEMBL27 were calculated based on the ECFP4 in the KNIME program [25]. The Tanimoto coefficients (Tc) [26] were employed to compare the chemical similarity between two molecules, ranging from 0 (completely dissimilar) to 1 (identical). For each of the five hits (Compounds 3, 7, 8, 12, and 15), the maximum Tc value compared to all of the known CB2 receptor antagonists in ChEMBL27 is presented in Table 4. The results in Table 4 show that the Tc values of the five hits ranged from 0.26 to 0.55. Compound 3, 7, 12, and 15, with the methanone (Figure S5A), pyrido[4′,3′:4,5]thieno[2,3-d]pyrimidine-2,4(1H,3H)-dione (Figure S5B), 4(3H)-pteridinone (Figure S5D) and 1H-pyrazole-5-carboxamide (Figure S5E) scaffold, respectively, had Tc values of 0.32, 0.28, 0.26 and 0.55, respectively. Compound 8 with the 4H-pyrido[1,2-a]pyrimidin-4-one scaffold (Figure S5C), with the highest binding affinity and the most potent antagonistic activity for the CB2 receptor, has a low Tc value of 0.37. Hence, the novel chemotypes of these compounds identified from multi-step virtual screening could serve as potential leads for CB2 drugs’ development.

### 2.6. Binding Mode of Compounds 3, 7, 8, 12, and 15 in the CB2 Receptor

**Figure 4:**
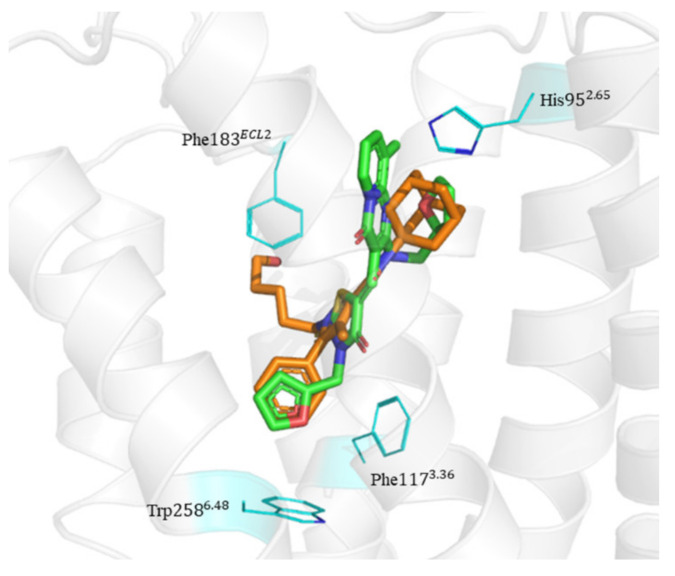
The binding mode of Compound 8 in the orthosteric site of CB2 receptor. Protein, AM10257, and Compound 8 are shown as a gray cartoon, orange stick, and green stick, respectively. The side chains of His95^2.65^, Phe117^3.36^, Phe183^ECL2^, and Trp258^6.48^ are represented as lines in blue color.

The binding mode of Compounds 3, 7, 8, 12, and 15 in the orthosteric site of the CB2 receptor was identified at the Glide XP docking level (Figure S6 and Figure 4). Phenylsulfonyl and 2-methoxyphenyl of Compound 3 interact with Phe183^ECL2^ and Trp258^6.48^, respectively, through π–π stacking (Figure S6A). The 2-chloro-6-fluorophenyl of Compound 7 interacts with Phe183^ECL2^ and Trp194^5.43^ through π–π stacking, and Pyrido[4′,3′:4,5]thieno[2,3-d]pyrimidine-2,4(1H,3H)-dione and phenylmethyl interact with Phe183^ECL2^ and Trp258^6.48^ through π–π stacking, respectively (Figure S6B). The 4(3H)-Pteridinone of Compound 12 interacts with Phe87^2.57^ and Phe183^ECL2^ through π–π stacking and interacts with Ser285^7.39^ through a hydrogen bond. The 3-fluorophenyl of Compound 12 interacts with Phe94^2.64^ and Phe106^3.25^ through π–π stacking (Figure S6C). The 4-fluorophenyl of Compound 15 interacts with Phe117^3.36^, Trp194^5.43^, and Trp258^6.48^ through π–π stacking, and thienyl interacts with Trp194^5.43^ through π–π stacking (Figure S6D). The binding mode of Compound 8 against the CB2 receptor is shown in Figure 4. We can see from Figure 4 that in the binding pocket of the CB2 receptor, the furan ring and 4H-pyrido[1,2-a]pyrimidin-4-one of Compound 8 interacts with Phe117^3.36^, Phe183^ECL2^, and Trp258^6.48^ through π–π stacking, and 4H-pyrido[1,2-a]pyrimidin-4-one of Compound 8 interacts with His95^2.65^ through π–cation interaction. These interactions are consistent with the key interactions of AM10257 in the orthosteric sites [14]. The binding pose of Compound 8 aligns well with AM10257 in the binding pocket.

## 3. Materials and Methods

### 3.1. Data Sets

To generate the DNN and CNN models, the known antagonists of CB2 receptor were downloaded from the ChEMBL27 database [18] and then processed by KNIME software [27]. The compounds with pK_i_ greater than 8 were regarded as active, while the compounds with pK_i_ less than 6 were considered as inactive. A total of 1573 compounds, including 859 actives and 714 inactives, were split into training set, test set, and validation set with a ratio of 7:2:1.

For the pharmacophore model, 589 compounds with K_i_ values less than 5 nM were divided into 8 clusters through the Canvas Similarity and Clustering module (Canvas, Schrödinger, LLC, New York, NY, US), and 8 representative compounds were selected to form the training set. A validation set containing 29 antagonists with K_i_ values less than 5 nM and 858 decoys was used to evaluate the pharmacophore model. The decoys were generated by using the DecoyFinder [28] to search the ZINC database [29].

The 2D structures of the compounds in the training set, validation set, and ChemDiv library were converted to 3D structures with hydrogen atoms added, tautomers, and stereoisomers generated by using Ligprep (version 10.2, Schrödinger, LLC, New York, NY, US) with the OPLS-2005 force field [30]. The protonation states of the ligands were generated at pH =7.0 ± 2.0 using Epik (version 4.3, Schrödinger, LLC, New York, NY, US).

### 3.2. DNN and CNN Models

The DNN and CNN models were generated on the basis of the ECFP4 and NFP of the training set, respectively. The models were created and optimized using the Lasagne library of Theano [31]. To prevent the overfitting of the models, L2 regularization and Dropout were used. Rectified linear unit (ReLU) [32] and adaptive moment estimation (Adam) [33] were, respectively, used as the activation function and optimization algorithm of the models. Hyperparameters were optimized through random search. The learning rate, batch size, and L2 regularization in the DNN model are 0.001, 300, and 0.0001, respectively. The learning rate, batch size, and L2 regularization in the CNN model are 0.001, 350, and 0.0001, respectively. The performance of the models was evaluated using 6 indicators: sensitivity (SE) [34], specificity (SP) [34], prediction accuracy of active molecules (Q+), prediction accuracy of inactive molecules (Q−), Matthews’ correlation coefficient (MCC) [35], and the area under the receiver operating characteristic (AUC). The evaluation indicators were calculated as follows [36]:

(1) $$\mathrm{SE}=\frac{TP}{TP+FN}$$

(2) $$\mathrm{SP}=\frac{TN}{TN+FP}$$

(3) $$Q+=\frac{TP}{TP+FP}$$

(4) $$Q-=\frac{TN}{TN+FN}$$

(5) $$\mathrm{MCC}=\frac{TP\times TN-FN\times FP}{\sqrt{\left(TP+FN\right)\left(TP+FP\right)\left(TN+FN\right)\left(TN+FP\right)}}$$

where *TP*, *FN*, *TN*, and *FP* are the number of true positives, false negatives, true negatives, and false positives, respectively. The data sets and code of the DNN and CNN models can be downloaded through https://github.com/Houshujing/DNN-CNN-models, accessed on 3 November 2021 [37].

### 3.3. Pharmacophore Modeling

The multi-ligand pharmacophore model of CB2 receptor antagonists was constructed with the Phase module (version 5.4, Schrödinger, LLC, New York, NY, US). Eight compounds (Table S1) with K_i_ value less than 5 nM were used to generate the multi-ligand pharmacophore model through Develop pharmacophore model. A validation set containing 29 antagonists and 858 decoys was used to verify the screening performance of the pharmacophore model.

### 3.4. Molecular Docking

Glide module of Schrödinger [38,39] was used for molecular docking calculations. Crystal structures of the CB2 receptor with 2.80 Å resolution (PDB ID: 5ZTY) were downloaded from the RCSB Protein Data Bank (PDB) [40]. The Protein Preparation Wizard module was used to add hydrogens, fill in missing side chains and loops, and delete waters. The protonation status of hydroxyl, Asn, Gln, and His were assigned by using the ProtAssign module [41]. The CB2 receptor was then minimized using the OPLS-2005 force field [30] with converged root-mean-square deviation (RMSD) of the heavy atoms of 0.30 Å. Docking grid was generated using Glide default settings to define the center of the grid box with the co crystal ligand AM10257.

The Virtual Screening Workflow (version 7.8, Schrödinger, LLC, New York, NY, US) was used for docking screening, which includes three sequential docking steps: Glide HTVS, SP, and XP. At each step, the top 25% scoring compounds were further filtered by the next step. After Glide XP docking, the top 199 ranked compounds were retained for visual inspection. Finally, 15 compounds were selected for further biological activity evaluation.

### 3.5. Radioligand Binding Assays

The 15 compounds were purchased from J&K Scientific Ltd. (Shanghai, China). The radioligand binding assays and functional assays to CB2 receptor were performed by Pharmaron (Beijing) upon commission. In the CB2 receptor assays, [^3^H]-CP55940 was used as the radioligand; the 15 compounds were tested in radioligand binding assays at human CB2 receptor ([^3^H]-CP55940). The competition binding experiments of the test compounds at ten different concentrations and [^3^H]-CP55940 (0.8 nM) against CB2 receptor (2 μg/well) were performed for 90 min at 30 °C in 500 μL of assay buffer (25 mM HEPES, 10 mM MgCl_2_, 1 mM CaCl_2_, 0.5% BSA, pH 7.4).

### 3.6. Functional Assay

In this work, the 7 compounds with the best CB2 receptor affinity from the binding assays were selected to perform the functional assays. The CB2 receptor antagonistic activities of the 7 compounds were evaluated by investigating CB2-mediated cAMP production in Flpin-CHO-K1 cells functional assay. The Flpin-CHO-K1 cells that are engineered to stably express human CB2 receptor (the Flpin-CHO-CB2 cells) were cultured in the growth medium (Ham’s F12K + 10%FBS + 1×Ps + 800 ug/mL HB) at 37 °C and 5% CO_2_. The cells were then collected by centrifugation and resuspended in Hank’s balanced salt solution (HBSS), 0.1% bovine serum albumin (BSA), 20 mM N-(2-hydroxyethyl)-piperazine-N0-ethanesulfonic acid (HEPES), and 500 μM 3-isobutyl-1-methylxanthine (IBMX). The test compounds were serially diluted in DMSO at 2-fold dilutions of 10 concentrations ranging from 10,000 μM to 20 μM. The antagonistic activities were evaluated by assessing their ability to counteract the agonist (CP55940)-mediated decrease in cAMP accumulation. The accumulation of cAMP was determined by following the manufacturer’s instructions for a Perkin Elmer cAMP kit.

## 4. Conclusions

In this study, the ChemDiv database was screened to discover CB2 receptor antagonists using a multi-step virtual screening strategy including deep learning (DNN and CNN), pharmacophore, and molecular docking. Among the final hits, 15 compounds were selected to perform further bioassays. Among the 15 selected and tested compounds in the radioligand binding assays against CB2 receptor, 13 compounds were found to exhibit binding affinity, among which 7 compounds possessed good binding affinities with a pK_i_ of 5.15–6.66. In particular, Compound 8 has an affinity of nanomolar (pK_i_ = 6.66). Five of the seven compounds with good binding affinities in the radioligand binding assays were further tested in the cAMP functional assays and showed antagonistic activity against the CB2 receptor with a pIC_50_ of 5.25–6.93. Notably, Compound 8 shows the highest binding affinity for the CB2 receptor with a pK_i_ value of 6.66, the most potent antagonistic activity towards the CB2 receptor with a pIC_50_ value of 6.93, more than 100 times selectivity for the CB2 receptor over the CB1 receptor, and acceptable novelty with a Tc value of 0.37. Additionally, the docking results show that Compound 8 interacts with residues His95^2.65^, Phe117^3.36^, Phe183^ECL2^, and Trp258^6.48^ in the CB2 orthosteric site. Therefore, Compound 8 with the 4H-pyrido[1,2-a]pyrimidin-4-one scaffold could serve as a lead for further development of CB2 drugs. The developed approach could also be used to design potent ligands for other therapeutic targets.

## Figures and Tables

**Table 1:** Test results of the DNN classification models under different batch sizes.

DNN Model	Batch Size	SE	SP	Q+	Q−	MCC	AUC
Model_D1	50	0.968	0.903	0.911	0.966	0.874	0.982
Model_D2	100	0.975	0.897	0.906	0.972	0.875	0.98
Model_D3	150	0.956	0.942	0.944	0.954	0.898	0.982
Model_D4	200	0.956	0.923	0.926	0.953	0.879	0.982
Model_D5	250	0.949	0.903	0.909	0.946	0.854	0.981
Model_D6	300	0.975	0.942	0.945	0.973	0.917	0.986
Model_D7	350	0.975	0.923	0.928	0.973	0.899	0.982

**Table 2:** Test results of the CNN classification models under different batch sizes.

CNN Model	Batch Size	SE	SP	Q+	Q−	MCC	AUC
Model_C1	50	0.962	0.877	0.889	0.958	0.843	0.975
Model_C2	100	0.93	0.877	0.886	0.925	0.809	0.971
Model_C3	150	0.956	0.897	0.904	0.952	0.854	0.968
Model_C4	200	0.937	0.89	0.897	0.932	0.828	0.952
Model_C5	250	0.918	0.916	0.918	0.916	0.834	0.966
Model_C6	300	0.937	0.884	0.892	0.932	0.822	0.96
Model_C7	350	0.943	0.916	0.920	0.940	0.860	0.967
Model_C8	400	0.93	0.897	0.902	0.927	0.828	0.963

**Table 3:** Validation of the pharmacophore hypotheses.

Hypothesis	PhaseHypoScore	EF1%	BEDROC (α-160.9)	ROC	AUAC	Total Actives	Ranked Actives	Matches
AAHHR_1	0.89	10.20	0.39	0.71	0.71	29	28	4 of 5
AAHHR_2	0.89	10.20	0.34	0.71	0.71	29	28	4 of 5
AHHHR_1	0.86	6.80	0.32	0.67	0.67	29	27	4 of 5
AAHHR_4	0.85	13.59	0.47	0.68	0.68	29	28	4 of 5
AAHHR_3	0.83	6.80	0.20	0.72	0.72	29	28	4 of 5
HHHR_1	0.82	3.40	0.22	0.43	0.66	29	13	4 of 4
HHHR_2	0.81	10.20	0.30	0.43	0.67	29	13	4 of 4
AHHHR_2	0.79	6.80	0.31	0.68	0.69	29	27	4 of 5
AHHR_6	0.78	10.20	0.42	0.73	0.77	29	24	4 of 4
AHHR_1	0.77	13.59	0.39	0.64	0.70	29	22	4 of 4
AHHR_2	0.76	3.40	0.14	0.52	0.59	29	21	4 of 4
AHHHR_3	0.75	10.20	0.36	0.54	0.68	29	17	4 of 5
AHHHR_4	0.73	6.80	0.27	0.65	0.69	29	23	4 of 5
AHHR_7	0.72	0	0.01	0.60	0.68	29	21	4 of 4
AHHR_4	0.68	0	0.01	0.51	0.62	29	18	4 of 4
AHHR_3	0.68	0	0.00	0.55	0.65	29	19	4 of 4
AAHR_1	0.67	0	0.01	0.56	0.65	29	19	4 of 4
AHHHR_5	0.67	10.20	0.44	0.63	0.65	29	24	4 of 5
AHHR_5	0.67	6.80	0.41	0.49	0.68	29	15	4 of 4
AHHHR_6	0.66	6.80	0.31	0.62	0.64	29	25	4 of 5

**Table 4:** The chemical structures, the binding affinities (pK_i_) obtained from radioligand binding assays at human CB2 receptor, the potencies (pIC_50_) at human CB2 receptor, and the Tc values of the 15 hits.

Compound Number	Compound ID	Chemical Structures	Binding Affinities pK_i_	cAMP Assay pIC_50_	LogP	Tc
1	G748-0093	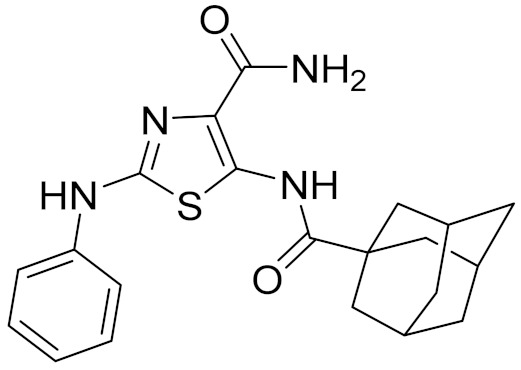	5.80	<4.70	3.73	
2	8018-1162	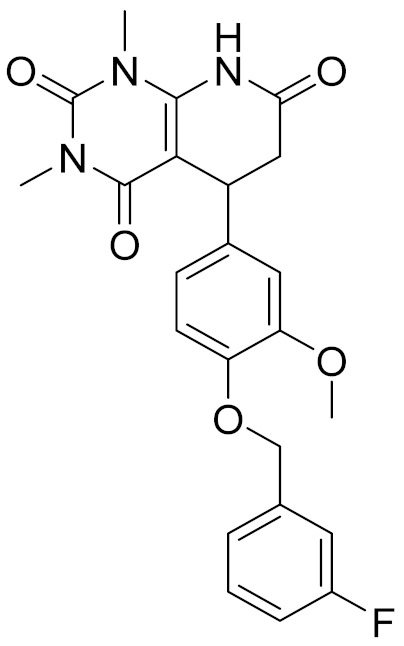	<4	-	3.53	
3	C200-3916	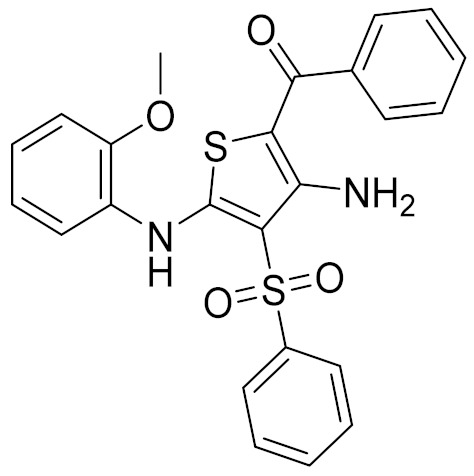	5.31	6.47	4.91	0.32
4	C688-1110	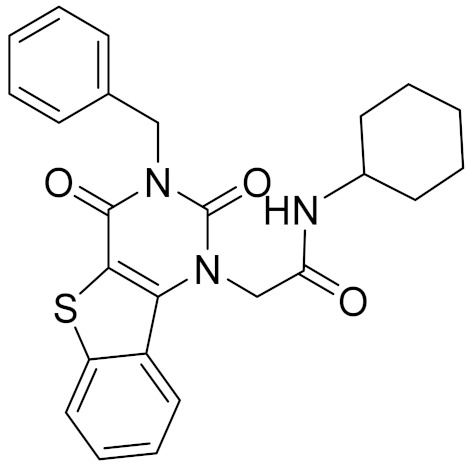	<4	-	5.58	
5	E196-0346	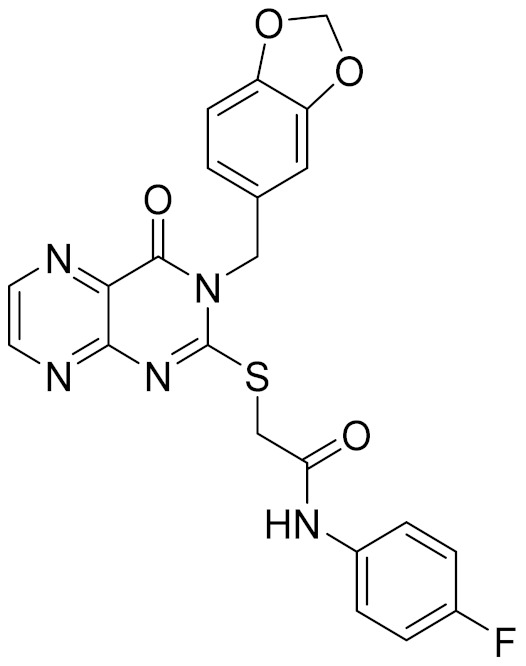	4.42	-	3.01	
6	C728-0838	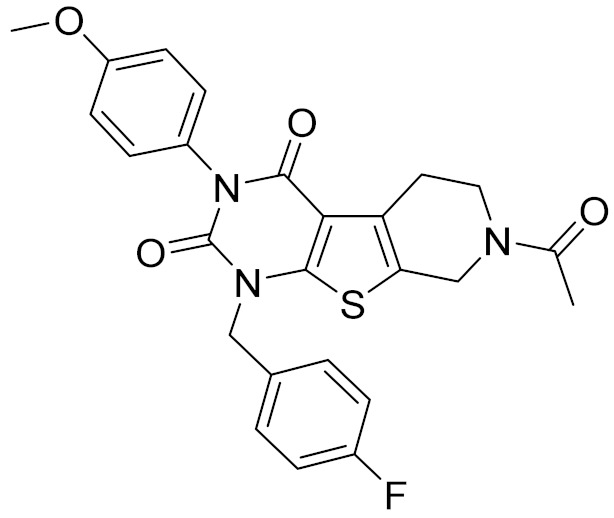	5.15	<4.70	3.07	
7	C728-0198	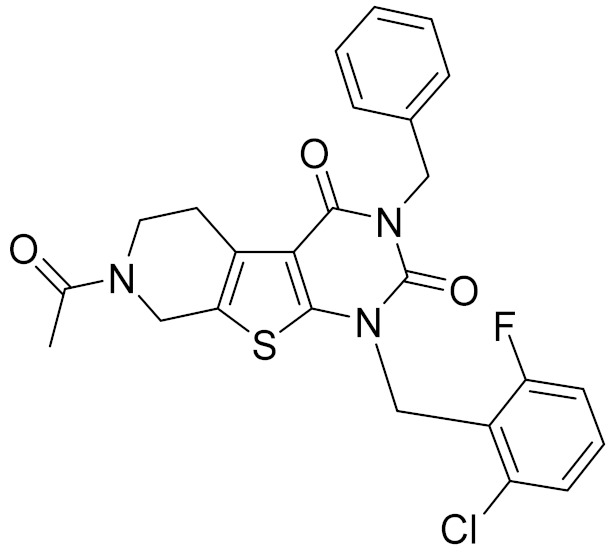	5.18	5.46	4.74	0.28
8	4428-0510	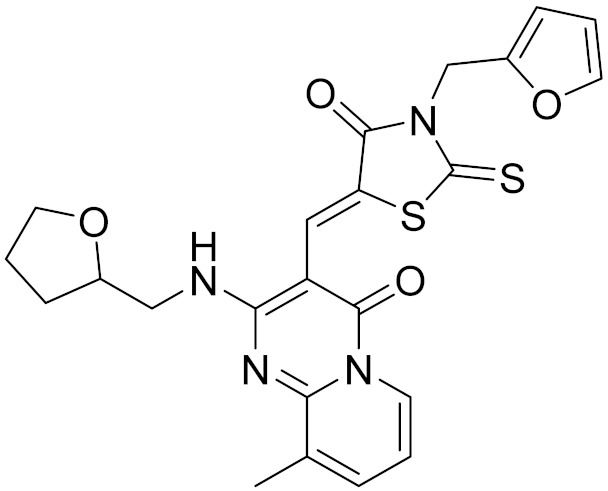	6.66	6.93	4.55	0.37
9	C566-1034	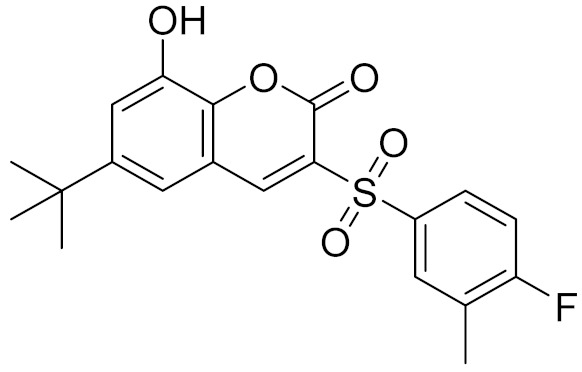	4.63	-	3.36	
10	C241-0788	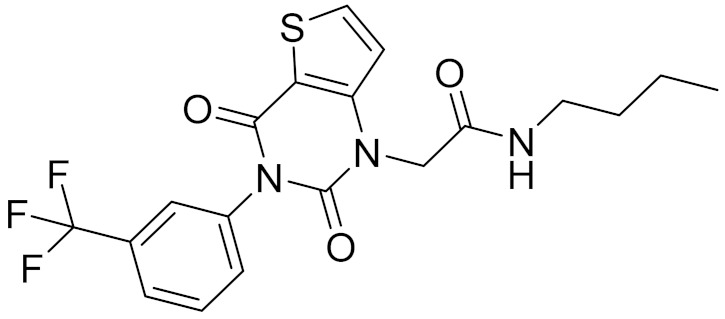	4.75	-	3.80	
11	E146-1216	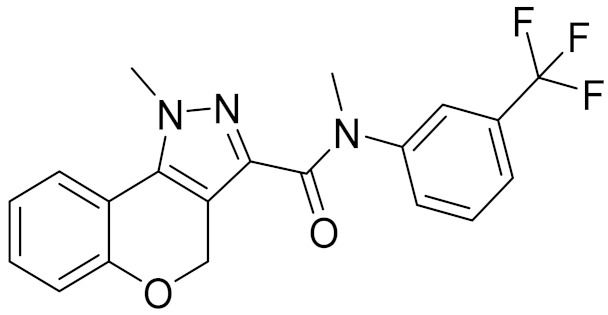	4.86	-	3.93	
12	E196-0403	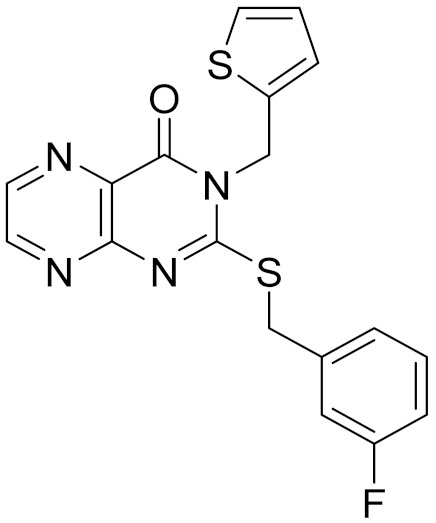	5.45	5.25	3.09	0.26
13	E538-0230	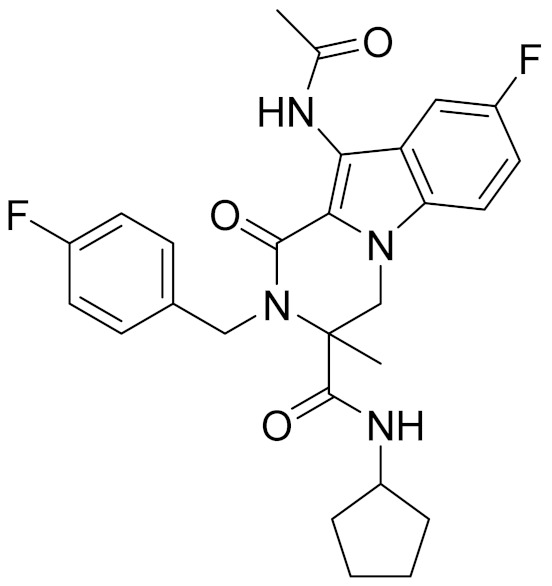	4.48	-	4.34	
14	C796-1142	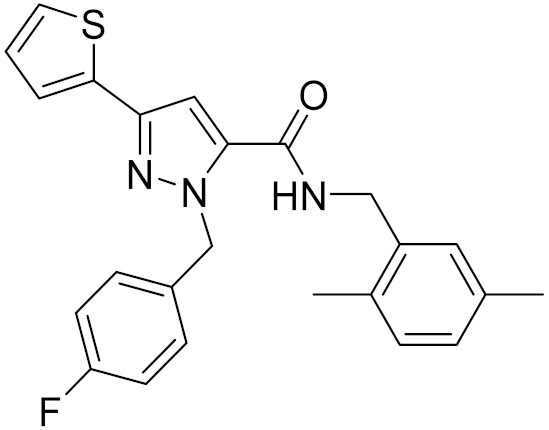	4.75	-	7.58	
15	C796-1158	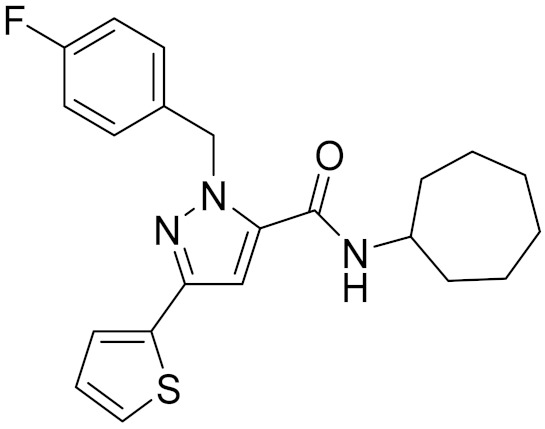	5.76	5.55	7.61	0.55
-	WIN-55212-2	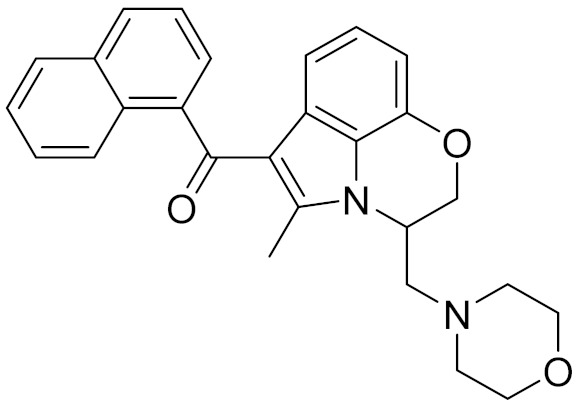	8.27	-	5.13	-
-	SR144528	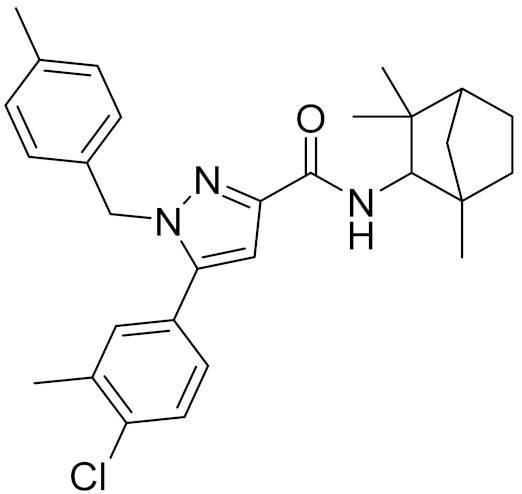	-	7.49	10.17	-

## Data Availability

The datasets analyzed during the current study are available in the ChemDiv (https://www.chemdiv.com/, accessed on 3 November 2021) and ChEMBL (https://www.ebi.ac.uk/chembl/, accessed on 3 November 2021) repositories. All codes are publicly available at https://github.com/Houshujing/DNN-CNN-models, accessed on 3 November 2021.

## Supplementary Materials

The following are available online. Table S1: Training set for the CB2 receptor antagonist pharmacophore models. Table S2: The binding affinities (pK_i_) obtained from radioligand binding assays at human CB1 receptor and the potencies (pIC_50_) at human CB1 receptor of the 5 compounds (Compounds 3, 7, 8, 12, and 15). Figure S1: (A–O): Concentration-response curves of compounds against CB2 receptor in the radioligand binding assays. Figure S2: (A–G): Concentration-response curves of compounds against CB2 receptor in the cAMP assay. Figure S3: (A–E): Concentration-response curves of compounds against CB1 receptor in the radioligand binding assays. Figure S4: (A–E): Concentration-response curves of compounds against CB1 receptor in the cAMP assay. Figure S5: Chemical structures of (A) Compound 3, (B) Compound 7, (C) Compound 8, (D) Compound 12, and (E) Compound 15. The scaffolds are shown as blue. Figure S6: The binding mode of (A) Compound 3, (B) Compound 7, (C) Compound 12, and (D) Compound 15 in the orthosteric site of CB2 receptor. The protein and compounds are shown as a gray cartoon and green stick, respectively. The side chains of Phe87^2.57^, Phe94^2.64^, Phe106^3.25^, Phe117^3.36^, Phe183^ECL2^, Trp194^5.43^, Trp258^6.48^, and Ser285^7.39^ are represented as lines in blue color.

## Sample Availability

All the mentioned compounds can be bought from either ChemDiv or other chemical suppliers.
