# Supplementary material for: Identification of Novel Antagonists Targeting Cannabinoid Receptor 2 Using a Multi-Step Virtual Screening Strategy
Source: Molecules. 2021 Nov 4;26(21):6679. doi: 10.3390/molecules26216679 (PMC8587544; doi:10.3390/molecules26216679)
Supplement: Supplementary file 1 [file molecules-26-06679-s001.zip › molecules-1412717-supplementary.pdf]

Supplementary Materials

Identification of Novel Antagonists Targeting Cannabinoid Receptor 2 Using a Multi-step Virtual Screening Strategy

Mukuo Wang [1], Shujing Hou [1], Ye Liu [1], Dongmei Li [1,*] and Jianping Lin [1,2,3,*]

[1] State Key Laboratory of Medicinal Chemical Biology, Tianjin Key Laboratory of Molecular Drug Research, College of Pharmacy, Nankai University, Haihe Education Park, 38 Tongyan Road, Tianjin 300350, China; wangmukuo@126.com (M.W.); 2120191123@mail.nankai.edu.cn (S.H.); 2120191125@mail.nankai.edu.cn (Y.L.)

[2] Biodesign Center, Tianjin Institute of Industrial Biotechnology, Chinese Academy of Sciences, 32 West 7th Avenue, Tianjin Airport Economic Area, Tianjin 300308, China

[3] Platform of Pharmaceutical Intelligence, Tianjin International Joint Academy of Biomedicine, Tianjin 300457, China

* Correspondence: dongmeili@nankai.edu.cn (D.L.); jianpinglin@nankai.edu.cn (J.L.)

Contents:

- **Table S1.** Training set for the CB2 receptor antagonist pharmacophore models.
- **Table S2.** The binding affinities (pK_i) obtained from radioligand binding assays at human CB1 receptor and the potencies (pIC_{50}) at human CB1 receptor of the 5 compounds (Compounds 3, 7, 8, 12 and 15).
- **Figure S1.** (A) – (O): Concentration-response curves of compounds against CB2 receptor in the radioligand binding assays.
- **Figure S2.** (A) – (G): Concentration-response curves of compounds against CB2 receptor in the cAMP assay.
- **Figure S3.** (A) – (E): Concentration-response curves of compounds against CB1 receptor in the radioligand binding assays.
- **Figure S4.** (A) – (E): Concentration-response curves of compounds against CB1 receptor in the cAMP assay.
- **Figure S5.** Chemical structures of (A) Compound 3, (B) Compound 7, (C) Compound 8, (D) Compound 12 and (E) Compound 15. The scaffolds are shown as blue.
- **Figure S6.** The binding mode of (A) Compound 3, (B) Compound 7, (C) Compound 12 and (D) Compound 15 in the orthosteric site of CB2 receptor. The protein and compounds are shown as a gray cartoon and green stick, respectively. The side chains of Phe87$^{2.57}$, Phe94$^{2.64}$, Phe106$^{3.25}$, Phe117$^{3.36}$, Phe183^{ECL2}, Trp194$^{5.43}$, Trp258$^{6.48}$ and Ser285$^{7.39}$ are represented as lines in blue color.

Table S1. Training set for the CB2 receptor antagonist pharmacophore models.

Compound number	Compound ID	Chemical structures	K_i (nM)
			CB2
1	CHEMBL108868	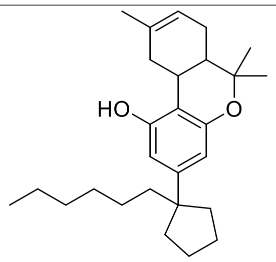	1.91
2	CHEMBL1076685	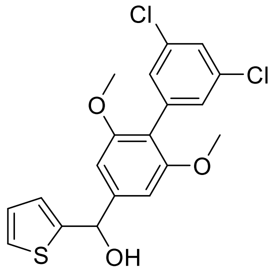	0.2

3	CHEMBL231719	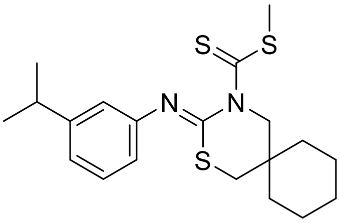	2.5
4	CHEMBL1668519	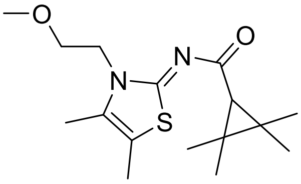	0.64
5	CHEMBL1162994	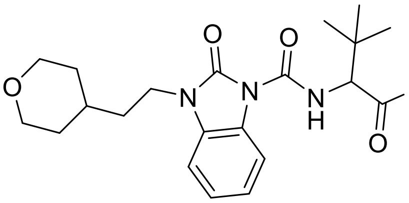	4
6	CHEMBL1215918	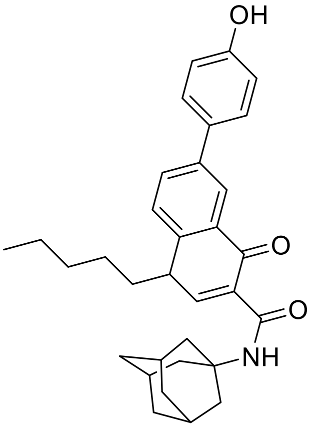	4.2
7	CHEMBL3909238	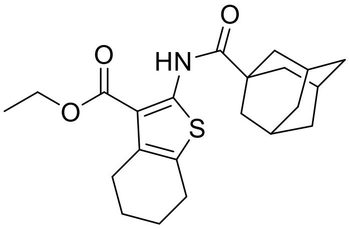	2.15
8	CHEMBL1270480	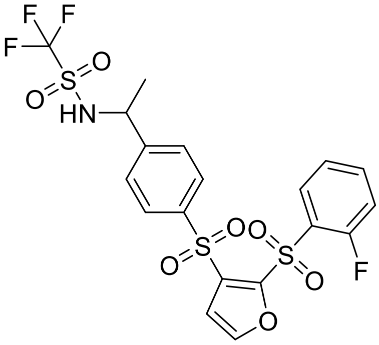	4.4

Table S2. The binding affinities (pK_i) obtained from radioligand binding assays at human CB1 receptor and the potencies (pIC_{50}) at human CB1 receptor of the 5 compounds (Compounds 3, 7, 8, 12 and 15).

Compound number	Compound ID	Binding affinities pK_i	cAMP assay pIC_{50}
3	C200-3916	4.73	<4.70
7	C728-0198	4.78	5.15
8	4428-0510	5.47	<4.70
12	E196-0403	4.42	<4.70
15	C796-1158	4.59	5.34

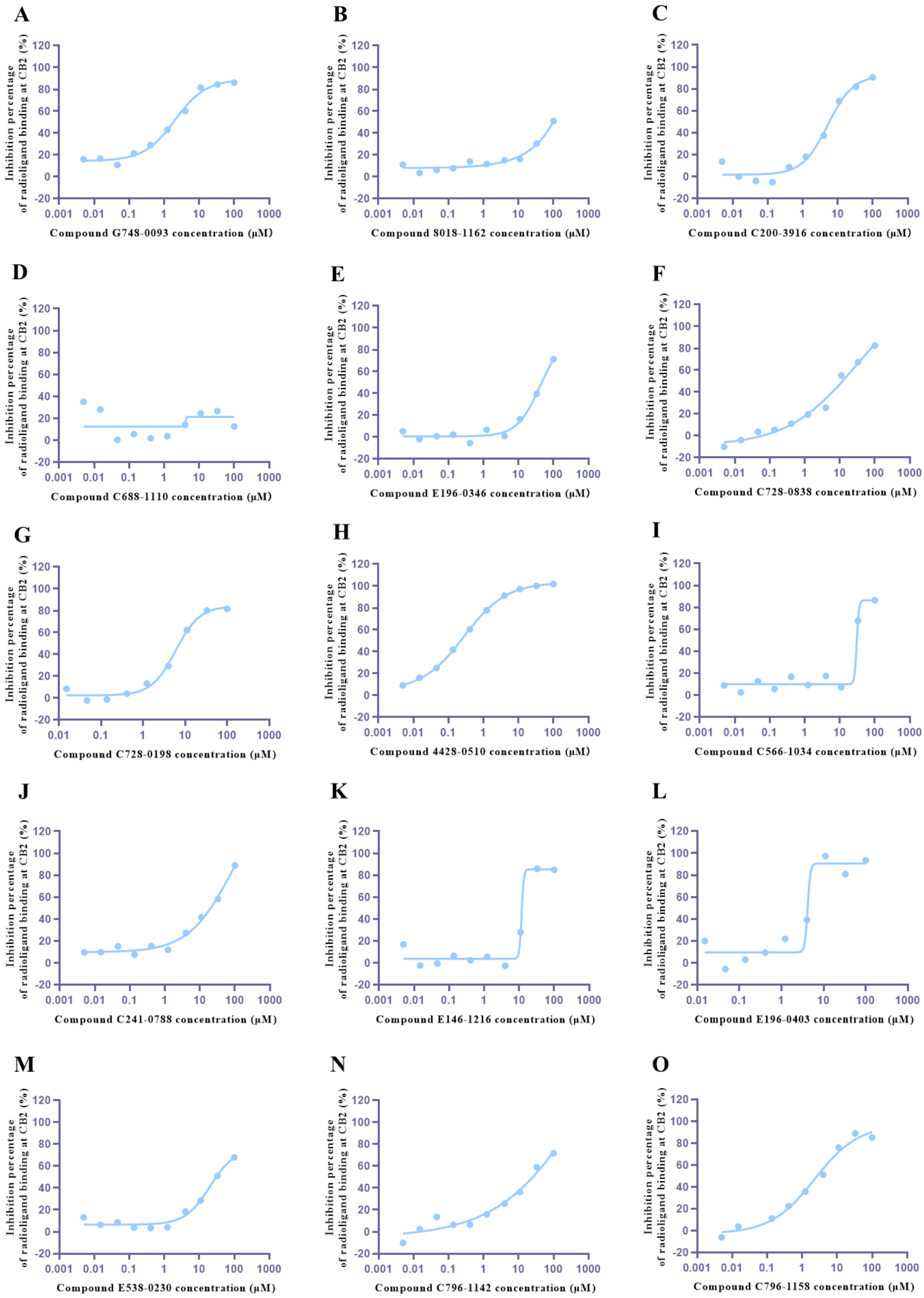

Figure S1. (A) – (O): Concentration-response curves of compounds against CB2 receptor in the radioligand binding assays.

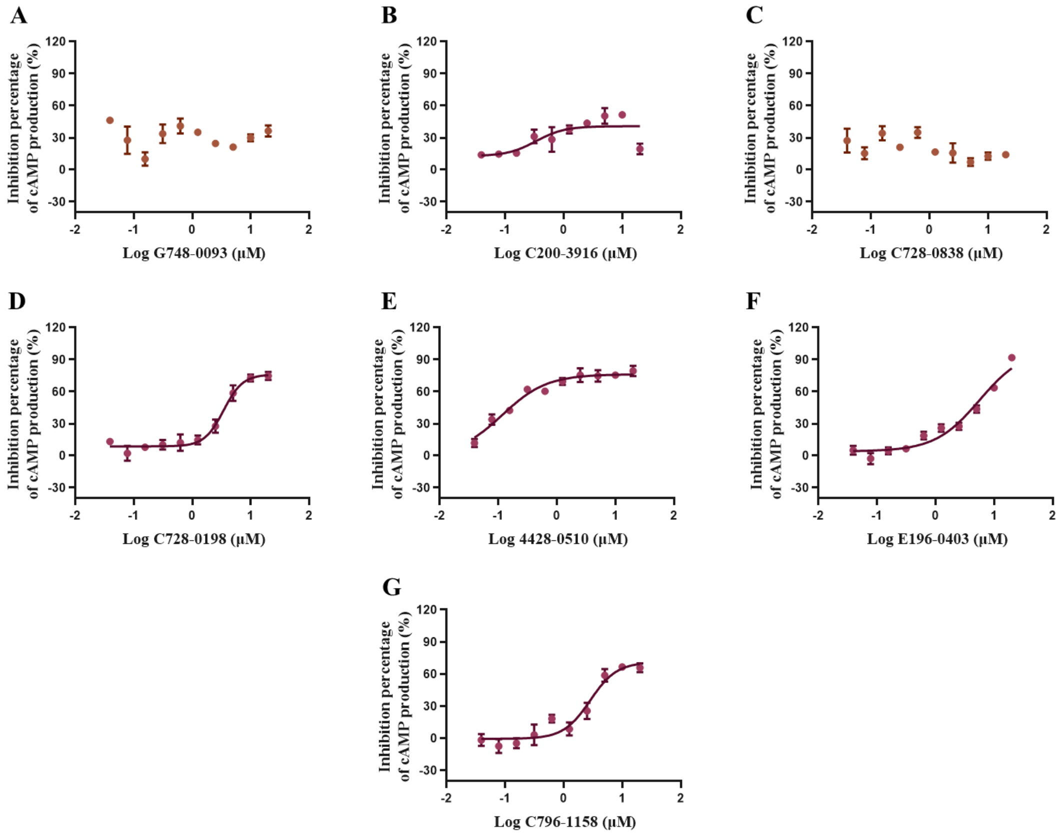

Figure S2. (A) – (G): Concentration-response curves of compounds against CB2 receptor in the cAMP assay.

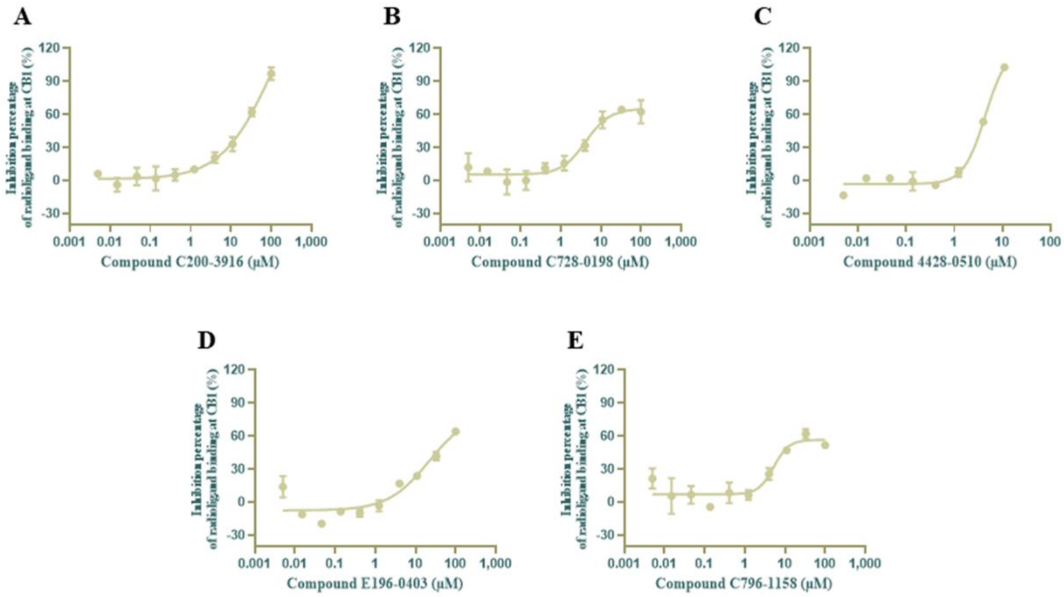

Figure S3. (A) – (E): Concentration-response curves of compounds against CB1 receptor in the radioligand binding assays.

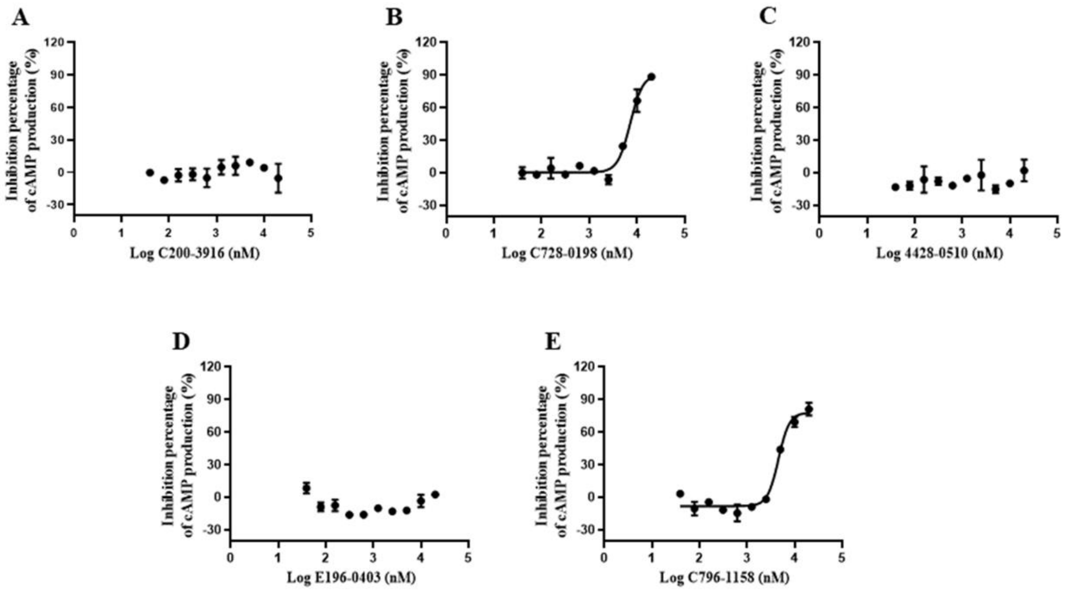

Figure S4. (A) – (E): Concentration-response curves of compounds against CB1 receptor in the cAMP assay.

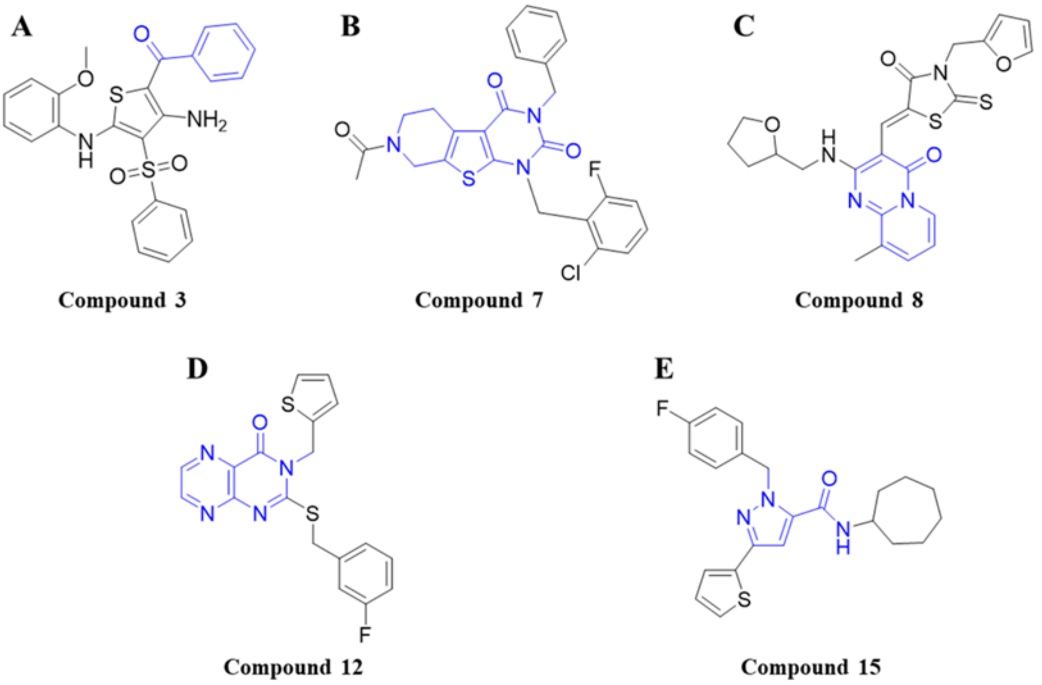

Figure S5. Chemical structures of (A) Compound 3, (B) Compound 7, (C) Compound 8, (D) Compound 12 and (E) Compound 15. The scaffolds are shown as blue.

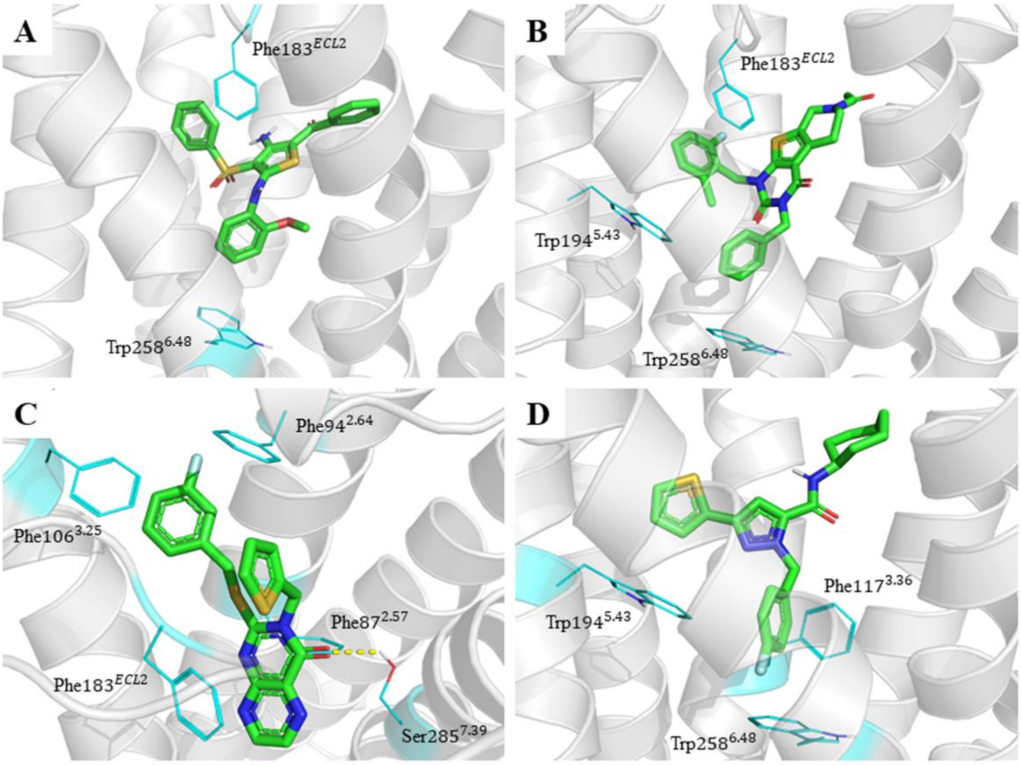

Figure S6. The binding mode of (A) Compound 3, (B) Compound 7, (C) Compound 12 and (D) Compound 15 in the orthosteric site of CB2 receptor. The protein and compounds are shown as a gray cartoon and green stick, respectively. The side chains of Phe87$^{2.57}$, Phe94$^{2.64}$, Phe106$^{3.25}$, Phe117$^{3.36}$, Phe183^{ECL2}, Trp194$^{5.43}$, Trp258$^{6.48}$ and Ser285$^{7.39}$ are represented as lines in blue color.
